# A nasal visual field advantage in interocular competition

**DOI:** 10.1038/s41598-022-08473-w

**Published:** 2022-03-17

**Authors:** A. Sahakian, C. L. E. Paffen, S. Van der Stigchel, S. Gayet

**Affiliations:** Experimental Psychology, Helmholtz Institute, Utrecht University, Heidelberglaan 1, 3584 CS Utrecht, The Netherlands

**Keywords:** Sensory processing, Visual system

## Abstract

When our eyes are confronted with discrepant images (yielding incompatible retinal inputs) *interocular competition* (IOC) is instigated. During IOC, one image temporarily dominates perception, while the other is suppressed. Many factors affecting IOC have been extensively examined. One factor that received surprisingly little attention, however, is the stimulus’ *visual hemifield* (VHF) of origin. This is remarkable, as the VHF location of stimuli is known to affect visual performance in various contexts. Prompted by exploratory analyses, we examined five independent datasets of breaking continuous flash suppression experiments, to establish the VHF’s role in IOC. We found that targets presented in nasal VHF locations broke through suppression much faster than targets in temporal VHF locations. Furthermore, we found that the magnitude of this nasal advantage depended on how strongly the targets were suppressed: the nasal advantage was larger for the recessive eye than for the dominant eye, and was larger in observers with a greater dominance imbalance between the eyes. Our findings suggest that the nasal advantage reported here originates in processing stages where IOC is resolved. Finally, we propose that a nasal advantage in IOC serves an adaptive role in human vision, as it can aid perception of partially occluded objects.

## Introduction

As our eyes are horizontally separated, visual information entering them leads to different projections on the two retinae. These two inputs are usually fused to form a single percept, and the slight difference between the projections can contribute to the extraction of depth information from the otherwise flat retinal projections. Fusing the inputs from the eyes to form a single coherent percept happens automatically and continuously, and falls outside of voluntary or conscious control (for reviews see Refs.^1,2^). When substantially different images are presented to corresponding retinal locations of the two eyes, however, the fusion of these images breaks down. Instead, the images are perceived in alternation; a phenomenon known as binocular rivalry^3,4^. The two incompatible images entering the visual system thus metaphorically compete to reach conscious perception: they engage in so-called interocular competition (IOC).

Experimental paradigms based on IOC have lent themselves as an excellent tool for many fields of research in vision and the cognitive sciences. On one end of the spectrum, IOC provides the means to investigate early stages of visual processing, where the information streams from the two eyes are still processed separately^5–7^. On the other end of the spectrum, IOC enables us to investigate the correlates of conscious perception, as it allows for changes in the observer’s conscious percept to occur, even though the visual input remains constant^8,9^. Evidently, paradigms based on IOC play an important role in understanding visual perception, from the earliest stages of processing visual input, up to and including the mechanisms governing the access to consciousness of visual stimuli.

**Figure 1:**
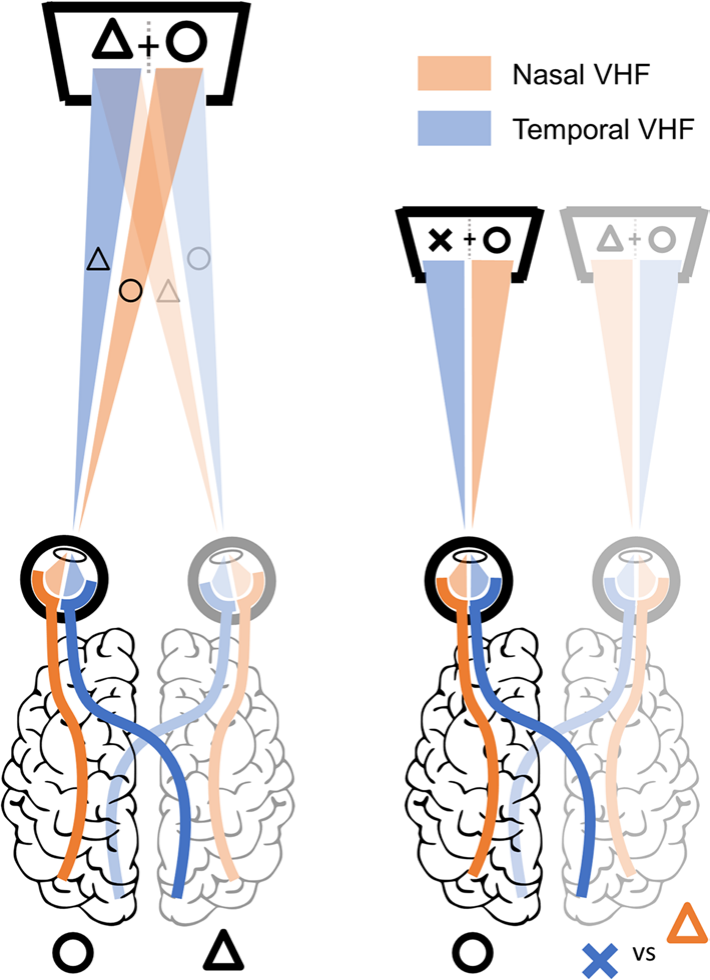
Schematic depiction of the path visual information follows from the visual hemifields to visual cortex. On the left a ‘normal’ viewing situation is depicted in which stimuli left and right of fixation (the triangle and circle, respectively) are projected to the opposite hemiretinae of both eyes. Information from the temporal (blue) *visual hemifields* (VHFs) is processed by the contralateral cerebral hemisphere, while information from the nasal (orange) VHFs is processed by the ipsilateral cerebral hemisphere. Usually, information from the two eyes ending up in one cerebral hemisphere matches and a coherent image is perceived. However, as depicted on the right, when conflicting information (presented dichoptically) ends up in one cerebral hemisphere, *interocular competition* (IOC) is evoked. Irrespective of whether conflicting information is present, visual information presented either left or right of fixation, is located in one eye’s nasal VHF and the other eye’s temporal VHF. In the case of IOC however, a stimulus in the nasal VHF of one eye will consequently compete with a stimulus in the temporal VHF of the other eye.

The first reports of IOC stem from centuries ago. An example of such a report is that by Dutour, who, in 1760, stuck yellow and blue disks of cloth onto opposite sides of a cardboard, which he held perpendicular to his face such that each eye would see one piece. Instead of the colors blending to green (as he expected) he saw either the yellow or blue or a piecemeal of the two colored cloths^10^ (see translation by O’Shea^11^). More recently, a wide range of paradigms based on IOC have been developed and used continuously since the 1950’s^12^. This large variety of paradigms include a range of visual stimulus types (e.g., static images, flashing patterns, 3D objects, etc.)^13–15^, and many different forms of behavioral and physiological measurements (e.g., response times, pupil sizes, EEG, fMRI, etc.)^7,16,17^. One aspect of IOC that has received limited attention is the role of the *visual hemifield* (VHF) to which stimuli are presented (see Fig. 1). This is surprising, given that numerous studies have shown large and systematic differences in visual performance for stimuli presented in different VHFs in the absence of IOC^18–22^ (for reviews see Refs.^23,24^). These asymmetries between VHFs (e.g., in contrast sensitivity, saccadic latency, attentional biases, etc.) raise the question whether interocular competitive strength is also dependent on the VHF to which the competing stimuli are presented. Not many studies examined VHF asymmetries in IOC, and the studies which have, have provided conflicting results: some studies reported an enhanced performance for stimuli in nasal VHF locations^25–27^ while others suggested little effect of VHF location or even an advantage for stimuli in temporal VHF locations^28–30^. The studies that observed VHF asymmetries in IOC (in either direction) do not elaborate much on potential explanations for the VHF asymmetry. Studies that found a temporal VHF advantage speculate about potential low level differences between VHFs (e.g., higher retinal cone density in the nasal hemiretina), whereas studies finding a nasal VHF advantage point to a (usually unspecified) high level origin for the asymmetry.

The goal of this study was to test whether interocular competitive strength differs between VHFs. More specifically, we investigated the difference in competitive strength between stimuli presented in the nasal VHFs (projected to the temporal hemiretina) and the temporal VHF (projected on the nasal hemiretina). Contrasting the nasal and temporal VHFs is particularly interesting in an IOC context, as visual information presented to one eye’s *nasal* VHF competes with visual information presented to the other eye’s *temporal* VHF (see Fig. 1).

**Figure 2:**
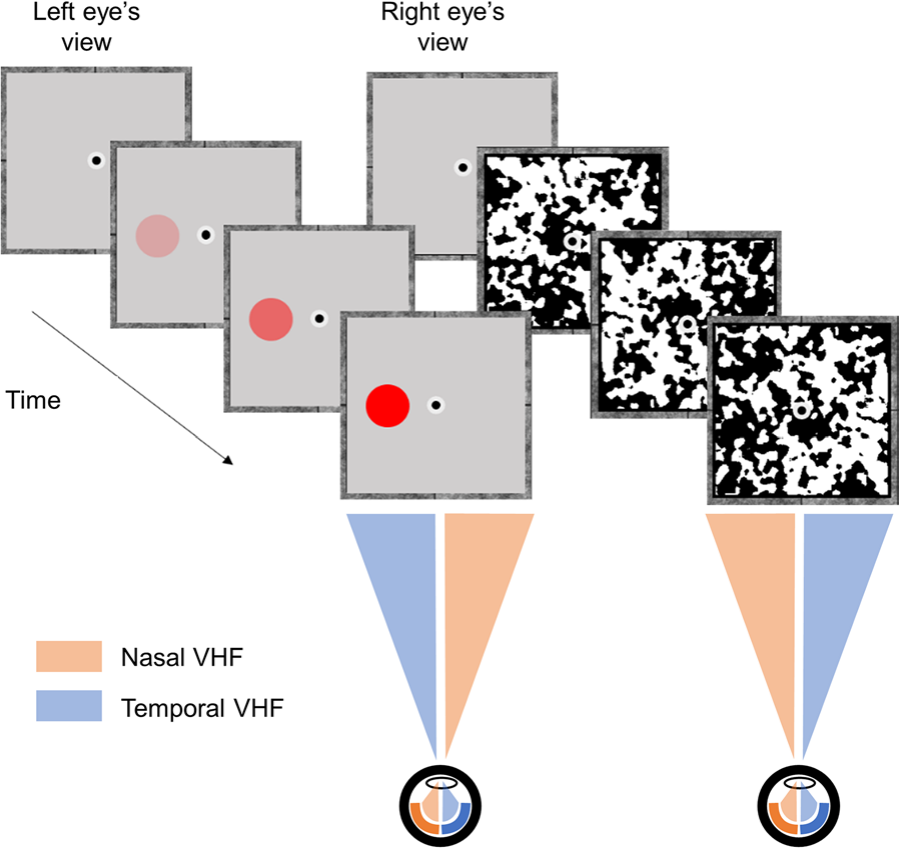
Overview of a single trial of a typical breaking Continuous Flash Suppression (b-CFS) experiment. A target is gradually introduced to one eye (the left eye in this example), while high-contrast masks are sequentially presented to the other eye (at ~ 10 Hz). Due to the much higher saliency of the masks relative to the target, the target will initially be suppressed from consciousness. Typically, after one, and up to several seconds, the target will overcome the suppression and will be perceived by the observer, who is then tasked with reporting one of its properties as fast as possible (which is often its location). In this example, the target is presented in the temporal VHF of the left eye and it is in competition with the part of the mask that is presented in the nasal VHF of the right eye.

We will focus on a particular IOC-based experimental paradigm called *breaking continuous flash suppression* (b-CFS). In this paradigm, a high-contrast flickering pattern (the mask) is presented to one eye, which initially suppresses a static stimulus (the target) that is gradually introduced to the other eye (see Fig. 2). The time it takes for observers to report some property of the initially suppressed target stimulus (e.g., its color or location), can be used as a proxy for the competitive strength of that target relative to the masks^31–33^. Continuous flash suppression (CFS), the masking technique underlying the b-CFS paradigm, has gained great popularity in the last decade as it provides a powerful means to render visual stimuli invisible for up to several seconds^14^, and allows for experimentally controlling which eye’s image is initially dominant. In the current study, we compared the suppression durations of targets presented in the nasal VHF and targets presented in the temporal VHF. We used data from five different studies using the b-CFS paradigm, conducted across three different laboratory setups of the authors. Following an exploratory analysis on an existing dataset—which triggered our initial interest for the current study, we expected to find a nasal VHF advantage in b-CFS response times. Importantly, this exploratory data set—described in more detail in Supplementary Text S1—is not included in any (confirmatory) analysis reported below.

To preface our results: We observe a strong effect of VHF on suppression durations, with nasally presented targets escaping suppression much faster than temporally presented targets; Expanding on this finding, we show that the nasal advantage (a) is specific to targets that are interocularly suppressed, (b) is largest for targets presented to the observers’ recessive eye, and covaries with individual observers’ eye-dominance strength. The relationship between the nasal advantage and the amount of interocular suppression (both within and between observer) suggests that the nasal advantage in b-CFS reflects a VHF asymmetry in IOC. In the discussion, we will tackle arguably the most intriguing question: *Why might there be a nasal advantage in IOC?* In answering this question, we discuss how a nasal advantage in IOC might serve to aid perception of partially occluded fixated objects.

## The nasal advantage

### The role of VHF in b-CFS

#### Methods

Our first goal was to test whether IOC strength depends on the VHF (i.e., nasal or temporal) to which a stimulus is presented. To this end, we analyzed datasets of previously conducted b-CFS experiments in our labs (Utrecht University, Experimental Psychology; Radboud University, Donders Institute). We included datasets on the basis of the following criteria:

1. The dataset must come from a b-CSF experiment. This meant that *observers were tasked to report as fast and accurately as possible a target stimulus’ location which was presented to one eye, while the other eye was presented with rapidly altering masking stimuli*.
2. Targets were presented to both the left and the right eye (on separate trials).
3. Targets were presented to both the left and right side of fixation (on separate trials).
4. Binocular stimulation was obtained using a mirror-stereoscope with either a single monitor set-up (preferably with a divider), or a two monitor set-up (one on each side of the participant), to effectively isolate the visual input of the two eyes.

These criteria lead to the inclusion of five complete datasets, with a combined total of 308 observers (see Table 1; for further details on each experiment see Supplementary Table S1). Due to anonymization of these datasets, however, we could not ascertain whether every observer in the combined dataset was a unique individual. Furthermore, there are two points to address about the experiments behind these datasets.

**Table 1:** Key aspects of the five main datasets analyzed in the current study.

Name and source of dataset	Number of observers	Trials per observer^b^	Description of targets (color, shape, size^a^, eccentricity^a^)	Mean (raw) RT, across observers	Mean accuracy, across observes
Dataset 1 (unpublished dataset, made available by author SG)	55	126	Grayscale spheres and cubes, either 0.85° or 1.70° in size, and ~ 4.5° from fixation	1559 ms (SD 592)	98.55% (SD 2.92)
Dataset 2 (unpublished dataset, made available by Yun Ding)	181	79	Red, green or blue, colored circles, 1.2° in size, 1.8° from fixation	1443 ms (SD 470)	95.53% (SD 7.33)
Dataset 3 (unpublished dataset, made available by Yun Ding)	24	68	1.2°; rectangular black and with gratings, oriented 45° clockwise or counterclockwise	3422 ms (SD 1288)	82.12% (SD 11.37)
Dataset 4 (Exp 3 in Ref.^35^)	27	137	Grayscale faces (2.2° × 3.6°), or rectangular cut-out of eye region 90 (2.2° × 3.6°). ~ 2.6° from fixation	2583 ms (SD 977)	95.6% (SD 6.3)
Dataset 5 (unpublished dataset, made available by author SG)	21	142	Triangles, circles and squares, ~ 1° in size, ~ 2° from fixation	1907 ms (SD 597)	98.94% (SD 1.75)

The five independent datasets, “Dataset 1” through “Dataset 5”, were used in “The role of VHF in b-CFS” to establish the nasal advantage first observed in the exploratory dataset, and in “The origin of the nasal advantage” to investigate the relation with eye-dominance. ^a^The size and eccentricity (i.e., distance from fixation point) of the targets are expressed in degrees of visual angle (°). ^b^This is the average number of included trials per observer within a dataset. The accuracy shows in what percentage of trials a valid response was given (e.g., reporting the correct target location, within acceptable times). Note that only trials with correct responses were included in our analyses.

First, we had no information about the gaze position of the observers in any experiment. The lack of knowledge on gaze position could potentially be problematic as a target’s nasal/temporal location depended on the gaze position. Nevertheless, we can be quite certain that targets presented to the left or right of the fixation point appeared in the left or right visual hemifield, respectively. Because, in order for a target to fall in the other visual hemifield than intended (e.g., a target is presented left of fixation, but appears right of the eye’s gaze position), participants would need to systematically shift their gaze to a location beyond the target. This is particularly improbable, considering that the task of localizing targets to the left or right of fixation incentivized participants to fixate centrally. We therefore consider it safe to assume that (despite not having tracked the eyes) the targets’ locations are labeled correctly.

Second, the mean response times (RTs) between experiments varied considerably, although the task (specifically, the b-CSF part of a trial) in all experiments was identical: “Indicate the location of a masked target as soon as you discern it”. Note however, that a number of factors are likely to alter overall RTs in a b-CFS paradigm: the discrepancy between the target and the masking stimuli, differences in set-up (e.g., screen brightness), and differences in secondary tasks (e.g., concurrent maintenance of information in working memory). These and other factors introduce systematic RT differences between the included data sets. We accounted for such systematic differences in RTs before statistical analysis, using a RT normalization procedure (see step F below).

In order to perform an all-encapsulating analysis on datasets collected in varying experimental designs, we applied a data standardization procedure consisting of the following steps:

A. We removed all trials with incorrectly reported targets, as determined by the design of the respective experiment. These could be, for example, trials in which the wrong target location was reported, or trials that ended before a response was recorded.
B. From the remaining trials, we extracted the following variables: a number identifying the dataset; a number identifying the unique observer; the eye to which the target was presented (i.e., left or right eye); the location to which the target was presented (left or right of fixation); and the response time (RT) in milliseconds.
C. We removed all trials where the RT was faster than 300 ms (e.g., reflecting random button presses, or anticipatory responses).
D. From the Eye of Target Presentation and Location of Target Presentation we derived the VHF of Target Presentation for every trial, thus constructing a new independent variable. For example: in a trial with the target presented to the Left Eye and Right of Fixation, the VHF of Target Presentation is ‘Nasal’; for Left Eye and Left of Fixation, the VHF of Target Presentation is ‘Temporal’.
E. We transformed the response times, using the natural logarithm (in order to transform the typical rightwards skewed response time distribution into a more normal-shaped distribution).
F. We normalized the (log-transformed) response times over each eye of each observer: We grouped together the trials by each eye of each observer. Next, we divided the RT of each trial by its group’s mean RT. This normalization step ensured that RTs for all eyes and all observers in the different experiments were of comparable magnitude, thus removing nuisance variance caused by overall RT differences between eyes and observers^32^.

This procedure provided us with RTs for nasally presented and for temporally presented targets per eye, for all observers. Moreover, due to the RT normalization step, the standardization procedure allowed us to pool the data of all observers across the five datasets for all statistical analyses. Where relevant we have also explicitly reported the statistics of each dataset separately. Note that while all statistical analyses reported in this study are performed on log-transformed and normalized response times, the RT measures in the figures and reported averages were back-transformed to ms (from the log-transformed RTs) for more intuitive interpretation.

To test the hypothesis that nasally presented targets were released from suppression faster than temporally presented targets, we performed a directional Bayesian paired-samples t-test. All Bayesian analyses were done using JASP version 0.14.1, and in all cases we used the default settings (importantly, for specifying the prior distributions), additionally we used the seed value 1 when applicable^34^.

#### Results

**Figure 3:**
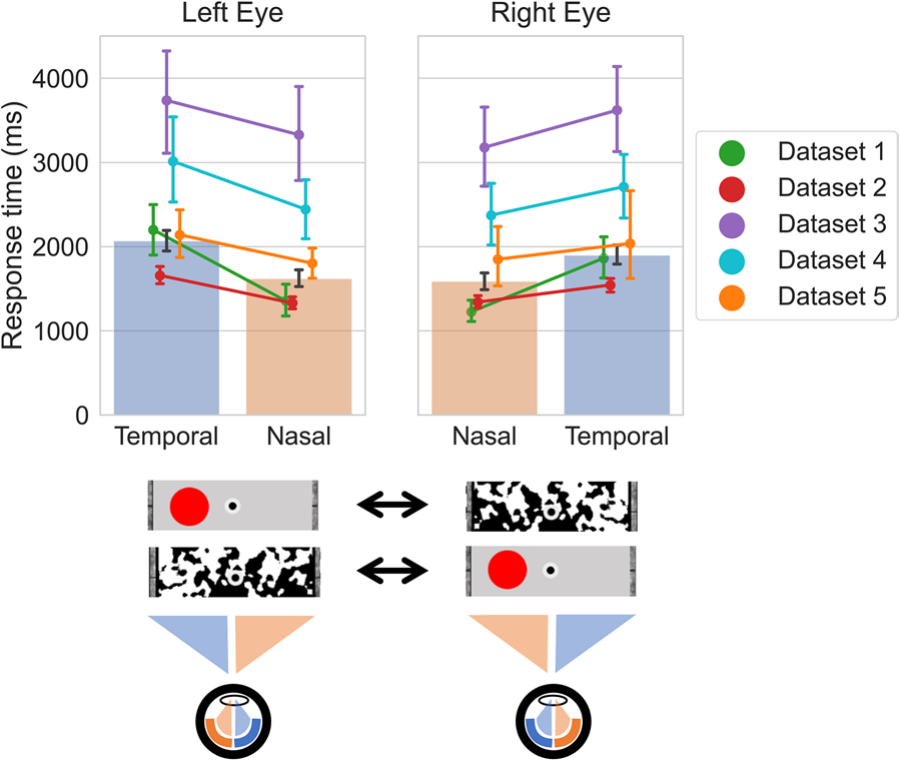
Response times per (left and right) eye per visual hemifield. Back-transformed response times to targets split over nasal and temporal presentation for the left and right eye. As illustrated in two examples below the graph (of the four possibilities), when a target (either nasal or temporal) is presented to one eye, it competes interocularly with a CFS mask presented to the other eye. The bars represent the data of all 308 included observers across the five datasets. The colored lines show the same data split over the five datasets. Error bars show the (bootstrapped) 95% confidence interval.

On average, across all datasets, observers took 1749 ms (SD 874) to respond to the initially suppressed targets. When we split the data over the VHF of target presentation, nasal targets were responded to in 1586 ms (SD 833), while temporal targets were responded to in 1956 ms (SD 993) (see Fig. 3).

A directional Bayesian Paired Samples *t*-test showed that the observed pattern of data was 3.96 × 10^44^ times more likely under our hypothesis (i.e., the RT measure for nasal targets is smaller than the RT measure for temporal targets) than under the null hypothesis (i.e., there is no difference in the RT measures for nasal and temporal targets). This indicates extreme evidential strength in favor of our hypothesis (see Fig. 3).

#### Interim discussion

The nasal VHF advantage reported above is an incredibly robust effect: the advantage is consistently and convincingly present in all five included datasets and for both eyes separately (all BFs_−0_ > 6.0; except in the Right Eyes in Dataset 5 where BF_−0_ = 1.206), with effect sizes (expressed as Cohen’s *d* values*)* ranging from 0.68 to 2.27. The difference in response times between the two VHF is particularly striking: nasally presented targets were responded to 370 ms (that is, 1.23 times) faster than temporally presented targets. The size and consistency of this effect suggest that this nasotemporal asymmetry might have a large influence on interocular competition.

An alternative possibility, however, is that the observed RT difference between nasal and temporal VHFs does not reflect a difference in IOC per se, but reflects differences in visual processing that emerge before the two monocular signals are combined (e.g., differences in contrast sensitivity between the nasal and temporal VHF). In the next section, we capitalize on a well-known asymmetry in IOC: the difference in IOC between the dominant and the recessive eye. Indeed in most observers, the input to one eye has a clear competitive advantage over input to the other eye, resulting in considerable response time differences in b-CFS paradigms between the dominant and recessive eye^36,37^. The rationale is that, if the nasal advantage observed in “Results” is related to IOC, then this advantage should be larger when there is more IOC and smaller when there is less IOC. Specifically, we predict that (1) the nasal advantage is larger in the recessive eye than in the dominant eye of individual observers, and that (2) observers with a larger difference between the dominant and the recessive eye should exhibit a stronger nasal advantage. To make our terminology explicit, when we speak of a nasal advantage in an eye (be it ‘the left eye’, ‘the dominant eye’, etc.), we refer to the fact that within this eye the targets presented in the nasal VHF break through suppression faster than targets presented in this eye’s temporal VHF.

### The origin of the nasal advantage

#### Methods: nasal advantage and eye dominance

To test our predictions about the role of IOC in the effect size of the nasal advantage, we again turned to the five datasets used in the previous section (Table 1), as this data already contained sufficient information to determine the sensory eye dominance of the observers. Although there are various ways to define eye dominance (which are not necessarily correlated^37^), we found sensory eye dominance the most suitable for our cause, as it (1) directly relates to IOC and (2) could be determined for all of our observers. Simply put, the eye of target presentation which elicited the fastest response times was labeled “dominant” and the other eye was labeled “recessive”. The modified data standardization procedure, which was unchanged up to and including step E, looked as follows (note that step G below, is essentially the same as step F in the previous procedure but relating to the dominant and recessive eye, instead of the left and right eye):

F. For each observer, we calculated the (log-transformed) mean RTs for each VHF of each eye. For each eye, we computed the average of the mean RTs of the two VHFs to get a mean RT for that eye. The eye with the fastest mean RT was labeled “Dominant” and the other eye was labeled “Recessive”. Thus, we constructed another independent variable, namely Eye (dominance) of Target Presentation.
G. We normalized the (log-transformed) response times over each eye of each observer: We grouped together the trials by each eye of each observer. Next, we divided the RT of each trial by its group’s mean RT. This normalization step ensured that RTs for all eyes and all observers in the different experiments were of comparable magnitude, thus removing nuisance variance caused by overall RT differences between eyes and observers^32^.

This procedure provided us with sufficient information to test our first prediction, that the nasal advantage is largest in the recessive eye. We performed a 2 × 2 Bayesian repeated measures ANOVA, with the RM factors of Eye (dominance) of Target Presentation and VHF of Target Presentation.

To test our second prediction (observers with a larger difference between the dominant and the recessive eye should exhibit a stronger nasal advantage), we needed to derive two new measures for each observer. On the one hand, we needed a measure to express which eye is dominant *and* to what extent; on the other hand, we needed a measure to express in which eye (left or right) the nasal advantage is largest *and* to what extent. We calculated the former measure by dividing the left eye’s mean (log-transformed) RT by the right eye’s mean (log-transformed) RT (see Eq. 1). We calculated the latter measure by first determining for each (left and right) eye the ratio between the temporal and nasal mean (log-transformed) RTs, then left eye’s ratio was divided by the right eye’s ratio to get the final measure (see Eqs. 2–4). We called these measures Dominance Ratio and Nasal Adv. Ratio respectively.

1 $$Dominance \; Ratio =\frac{ {RT}_{ Left \; eye}}{ {RT}_{ Right \; eye}},$$

2 $${Nasal \; Adv.}_{ Left \; eye}=\frac{{ RT}_{ Left \; eye \,\&\, Temporal \; VHF}}{{ RT}_{ Left \; eye \,\&\, Nasal \; VHF}},$$

3 $${Nasal \; Adv.}_{ Right \; eye}=\frac{{ RT}_{ Right \; eye\, \,\&\,\, Temporal \; VHF}}{{ RT}_{ Right \; eye \,\&\, Nasal \; VHF}},$$

4 $$Nasal \; Adv. \; Ratio =\frac{ {Nasal \; Adv.}_{ Left \; eye}}{ {Nasal \; Adv.}_{ Right \; eye}}.$$

We correlated the two resulting metrics across observers using a Bayesian Pearson correlation, to test whether observers with a higher dominance ratio (i.e., a more dominant right eye) also have a higher nasal advantage ratio (i.e., a larger nasal advantage in the right eye).

#### Results: nasal advantage in dominant versus recessive eye

We found that, on average, across all datasets, when the target was presented to individual observers’ dominant eye*,* they took 1616 ms (SD 814) to respond to the initially suppressed targets. Splitting the dominant eye’s RTs over the VHF of presentation revealed that nasally presented targets were responded to 311 ms (SD 557) faster (M = 1484 ms, SD 780) than temporally presented targets (M = 1795 ms, SD 966).

**Figure 4:**
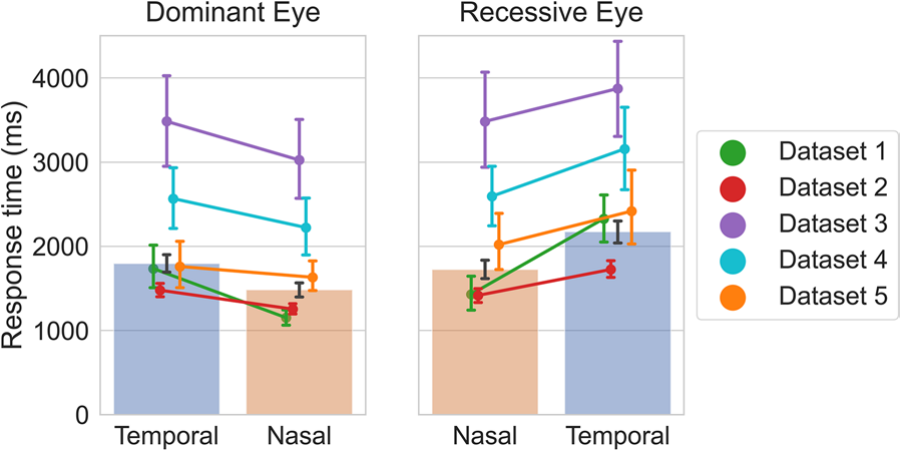
Response times per (dominant and recessive) eye per visual hemifield. Back-transformed response times to targets split over nasal and temporal presentation for both the dominant and recessive eye. The bars represent the data of all 308 included observers across the five datasets. The colored lines show the same data split over the five datasets. Error bars show the (bootstrapped) 95% confidence interval.

When the target was presented to observers’ recessive eye, observers took 1912 ms (SD 995) to respond. Note that the mean RTs for the recessive eyes is longer than that of the dominant eyes *by definition*. As we had labeled the eyes eliciting the shortest RTs as “dominant”. Splitting the recessive eye’s RTs over the VHF of presentation revealed that nasally presented targets were responded to 448 ms (SD 655) faster (M = 1724 ms, SD 973) than temporally presented targets (M = 2172 ms, SD 1138) (see Fig. 4).

The Bayesian RM ANOVA revealed strong evidence for the interaction between eye (dominance) of target presentation and VHF of target presentation, as supported by a Bayes Factor of 139.43 in the analysis of effects across matched models. This interaction indicates the VHF effect (i.e., nasal advantage) was (M = 138 ms, SD 731) larger in observers’ recessive eye than in their dominant eye.

It should be noted that the same data was used twice in this analysis; once for determining observers’ dominant eye (i.e., the eye for which targets led to the shortest RTs), and once for testing whether the nasal advantage was larger in the dominant eye compared to the recessive eye. We were concerned that for any random division of trials, the nasal advantage would be larger in the collection of trials that happen to contain longer RTs, because longer RTs are associated with larger RT differences^32,38^. Hence, a follow-up analysis was conducted to ensure that the difference in nasal advantage between the eyes did not spuriously emerge as a consequence of this ‘double dipping’ procedure. We randomly split each observer’s data in half, with the constraint that eye and VHF of target presentation remained balanced in both halves. Then, one half was used to label the eyes as ‘dominant’ and ‘recessive’, and the other half of the data was used to calculate the (normalized, as in step G) ratio in the nasal advantage size between the dominant and recessive eyes, which we named the *interaction value*. We repeated the procedure of randomly splitting and deriving an interaction value 10,000 times, and found that it was very unlikely that the interaction value would be 1 or lower (p = 0.0003) thus confirming the findings reported above.

A remarkable observation is that, across all observers in the datasets, the RT difference between nasally and temporally presented targets (Δ-VHF 370 ms, SD 475) was 1.25 times larger than the well-documented difference in RTs between the dominant and recessive eye (Δ-Eye Dominance 296 ms, SD 416).

#### Results: correlation of nasal advantage with eye dominance

**Figure 5:**
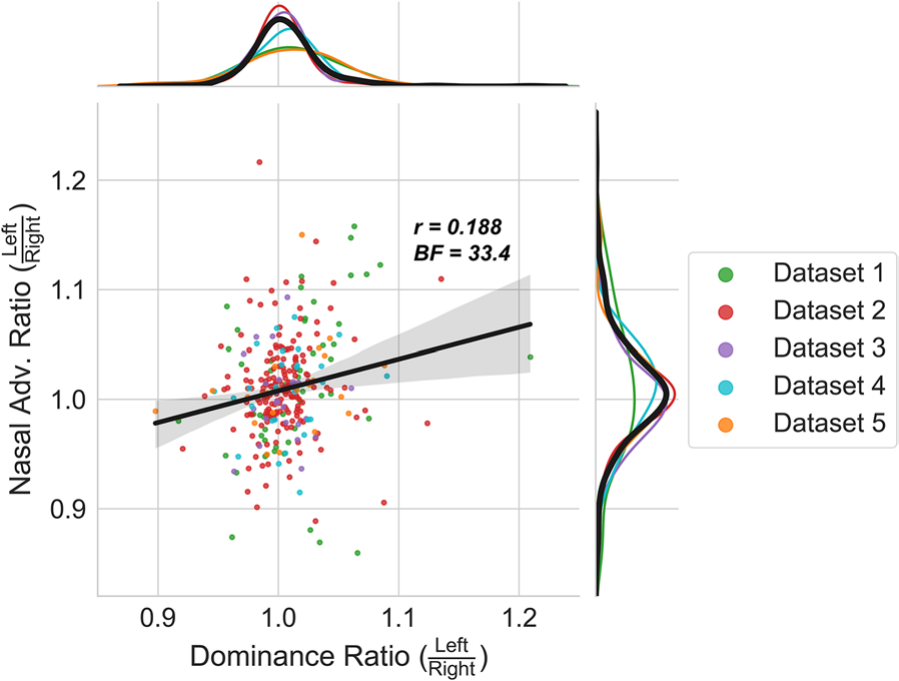
Correlation of the Dominance Ratio and the Nasal Adv. Ratio. Each dot represents the data of an individual observer. The correlation statistic and Bayes factor are computed with the pooled data of all observers across the five datasets. Different datasets are displayed in different colors. The smoothed distributions of the Dominance Ratios and the Nasal Adv. Ratios are placed on the upper and right border, respectively. The black line represents the data of all observers collapsed. The positive correlation indicates that participants with a larger imbalance between their dominant and recessive eye, tend to also exhibit a larger nasal advantages when targets are presented to their recessive eye compared to their dominant eye.

The Bayesian Pearson correlation resulted in a positive correlation between the Dominance Ratio and the Nasal advantage Ratio, with a Pearson’s r of 0.188, and a Bayes Factor of 33.36 (see Fig. 5). To ensure that the observed correlation was not driven by a single dataset, we computed the Pearson correlation five times leaving out only one dataset per iteration. We found that all *r* values were positive on every iteration: 0.115; 0.268; 0.197; 0.189 and 0.181 (with BF_+0_: 0.80; 21.7; 37.7; 23.3; and 16.7) when excluding dataset 1 through 5, respectively. As it seemed that Dataset 1 contributed most to the correlation, we should take some caution interpreting this result. Nevertheless, as (1) we did not base our conclusions solely on this correlation (but on two other analyses as well), and (2) an overall correlation can in principle be meaningful without a clear correlation in any single part of the whole, we believe it is justified to interpret the results of the overall correlation. This small—but reliable—positive correlation indicates that observers with larger dominance imbalance between eyes, show a larger difference in the nasal advantage between their eyes. The asymmetry in the nasal advantage is consistent with the direction of the imbalance: participants with a particularly dominant right eye tend to have a much weaker nasal advantage in the right eye compared to the left eye (and vice versa), and participants with no clearly dominant eye tend to have a comparable nasal advantage in both eyes.

#### Ruling out data processing artifacts

Next, we sought to ensure that the interaction between the nasal advantage and eye-dominance described above was real, and did not emerge as a result of our specific data preprocessing and analysis pipeline. To this end, we generated 10,000 simulated datasets, carefully matching the participant-level and population level RT distributions of the observed data. We also implemented all multivariate correlations between distributions that were present in the observed data (e.g., correlations between participants’ overall RT and RT difference between left and right targets, etc.). Importantly, the critical interaction between eye-dominance and the nasal advantage was *not* explicitly implemented in the simulated data, thus allowing us to test whether this interaction would emerge spuriously, as a result of our data analysis pipeline. We then applied our analysis pipeline to 10,000 simulated datasets, and computed the proportion of simulated datasets with an effect (i.e., a value expressing the interaction between eye-dominance and the nasal advantage) at least as large as the one observed in the experimental datasets. Reassuringly, we found of the 10,000 simulated datasets, not a single one had an effect equal or larger than the observed data had. We calculated the *z*-score of the observed effect to be 6.29 (i.e., the value was 6.29 SDs away from the mean of the distribution). We concluded that it was extremely unlikely that our data preprocessing and analysis pipeline gave rise to the critical interaction spuriously.

#### Monocular control condition

**Figure 6:**
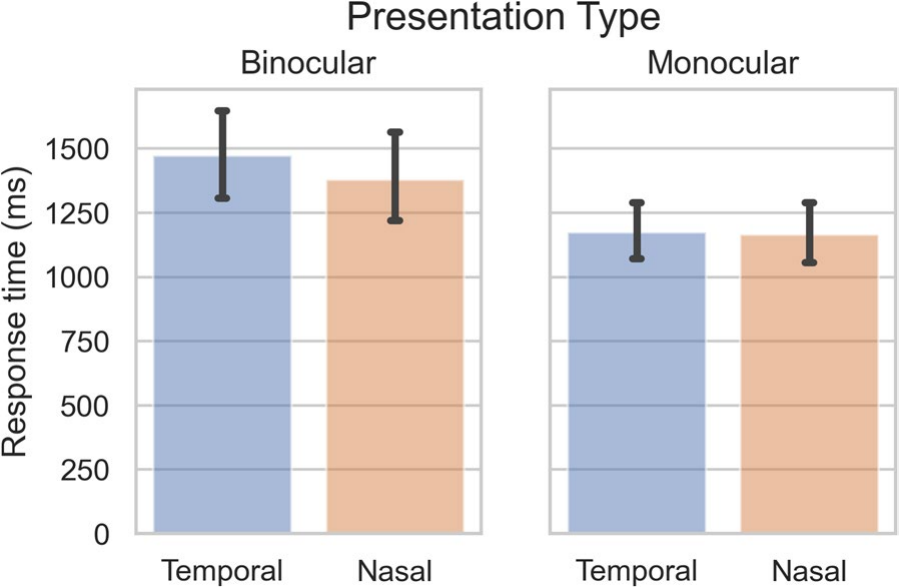
Response times with and without interocular competition. Response times of a b-CFS study with a monocular presentation condition (‘Dataset MC’ in Supplementary Table S1). The RTs measures for the VHFs of each observer were pooled across the two eyes. In the *Binocular* presentation condition the target and mask were presented to different eyes, inducing IOC. In the *Monocular* presentation condition the target (gradually increasing in contrast) and mask were presented to the same eye, so that no IOC was induced. Error bars represent (bootstrapped) 95% confidence intervals.

We performed one more test to confirm that the nasal advantage reported here actually pertains to a nasal advantage in IOC, rather than a nasal advantage occurring prior to or after IOC is resolved. We computed the nasal advantage in a new dataset (‘Dataset MC’ in Supplementary Table S1), in which the targets and the masks were either presented to *different eyes* (thus inducing IOC between masks and target; as was the case in the datasets analyzed so far) or to the *same eye* (thus not inducing IOC between masks and target). The results showed that the nasal advantage was observed in the condition with IOC (BF 3.64), but not in the (otherwise comparable) condition without IOC (BF 0.73; interaction BF 4.33) (see Fig. 6; for further details see Supplementary Text S2).

#### Ruling out response biases

**Figure 7:**
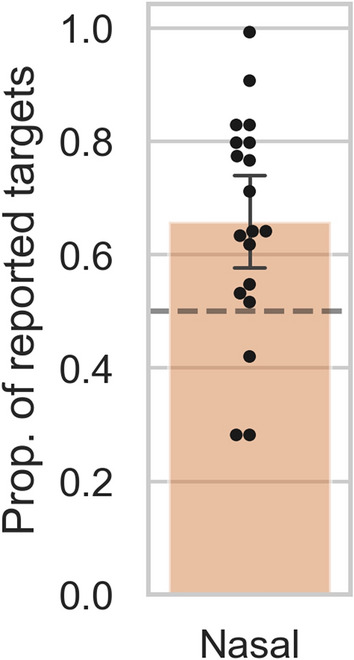
Proportion reported first perceived target locations. The proportion of reported targets which happened to be in a nasal or temporal VHF, averaged across observers (shown as dots in the plot). In this study’s b-CFS paradigm targets were presented both left and right of fixation (i.e., in a nasal and a temporal VHF) simultaneously, and observers reported which target appeared first. The dotted line represents the chance level, which was 0.5 as the two targets were always presented simultaneously. The error bar represents the (bootstrapped) 95% confidence interval.

Finally, we wanted to rule out that the nasal VHF advantage reported above reflected a difference in response tendency between the nasal and temporal hemifields, rather than the asymmetry reflecting a perceptual difference as we expected. One might argue that observers become aware of the nasal and temporal targets equally fast, but somehow (after becoming conscious) are biased to respond to nasal targets faster. An important idea to address here is that humans have, in principle, no conscious access to which eye is presented with what visual information^39,40^. That means, when a target and mask are presented to the two eyes separately, one does not know which eye is presented with the target, and which with the mask. Moreover, as the eye of target presentation was changing (unpredictably) on a trial-by-trial basis in the reported experiments, observers had no way of knowing to which of their two eyes a target was presented on any given trial. By extension, observers did not know (explicitly nor implicitly) whether a given target was in a nasal or temporal VHF. Therefore a response bias for targets in nasal VHF locations is unlikely to be responsible for the observed asymmetry. Nevertheless, we turned to a dataset from yet another b-CFS experiment, which allowed us to rule out such response bias empirically. In this experiment, two targets (one left and one right of fixation) were presented simultaneously. The observers’ task was to report the (left/right) location of the target that appeared first. We counted how often the reported “first appearing targets” happened to be presented in either the nasal or temporal VHF. Averaged across observers, in 66% (SD 20%) of trials nasal targets were reported to appear fist, which was substantially more often than expected by chance (BF_+0_ = 34.3; see Fig. 7). This finding shows that nasally presented stimuli are not only responded to faster, but are actually perceived earlier in time than temporally presented stimuli (For further details see Supplementary Text S3).

## General discussion

In this study we investigated the asymmetry in interocular competitive strength based on the visual hemifield (VHF) in which the stimuli are presented. Our interest in this topic was triggered by findings in an exploratory analysis and the lack of consensus in the limited pool of studies covering this topic. To investigate this potential asymmetry, we analyzed five independent datasets of previously conducted breaking continuous flash suppression (b-CFS) experiments. We found a relatively large advantage for nasally presented targets to break through interocular suppression, compared to temporally presented targets. Moreover, the advantage was convincingly present in all five datasets. The size and consistency of this VHF effect led us to hypothesize that the VHF of origin might play an important role in interocular competition (IOC). We tested three predictions that directly follow from the hypothesis that the VHF effect reflects an IOC-specific nasal advantage. These predictions were based on the assumption that—if the nasal advantage indeed originates during IOC—the VHF effect should depend on the amount of IOC, so that the nasal advantage is larger when there is more IOC. The first prediction was that the VHF effect would be larger when targets are presented in the recessive eye than in the dominant eye of observers. The second (related) prediction was that observers with larger imbalance in eye dominance, would show a larger difference in nasal advantage between their eyes. The third prediction was that the nasal advantage should only arise under conditions of IOC (when targets and masks are presented to different eyes), but not in the absence of IOC (when targets and masks are presented to the same eye). We found compelling evidence supporting all three predictions, suggesting that the VHF of origin indeed plays an important role in IOC. Furthermore, to preclude the possibility that the nasal advantage was driven by response bias, rather than being a perceptual effect, we analyzed one more data set, which showed that, regardless of response speed, nasal targets were reported to appear earlier in time than temporal targets when both were presented simultaneously.

How then do these findings relate to our current understanding of IOC and visual processing across VHFs? Considering first the research most comparable to our work here (i.e., investigating the nasotemporal asymmetry in IOC), we find little consensus in the handful of studies dedicated to this line of investigation. Whereas some studies show an advantage for the nasal VHF^25–27^, other studies showed either no, mixed, or even opposite results regarding the dominance of nasally versus temporally presented stimuli in IOC^28–30,41,42^. Regardless, the current study provides decisive results as we show a robust advantage for the nasal VHF during IOC evoked by b-CFS, across 300 + observers in five independent datasets. As there is little consensus on the nature of phenomenon itself, no effort (understandably) has yet been made to explain it. In the following paragraphs we discuss possible explanations for the diverging findings in the literature, we elaborate on our findings in greater detail, and we propose a functional explanation for the existence of a nasal advantage in IOC.

The first point we address is why previous studies evoking IOC report divergent results with respect to processes across VHFs. The first thing to note in this respect is that, in our current study, we have concerned ourselves exclusively with b-CFS. Interestingly, of the studies investigating nasotemporal asymmetries, the one using a b-CFS paradigm (as opposed to other studies in the literature using conventional BR measures) also finds a strong advantage for visual information in nasal VHF locations^27^. Why then, do the results from the two b-CFS studies differ from studies applying other paradigms evoking IOC (e.g., BR)? We suggest that different paradigms involving dichoptic presentation reveal different aspects of IOC, leading to different effects of VHFs. In support of this suggestion, it has been shown that, in the case of eye dominance, there is only a weak correlation between b-CFS and ongoing or onset rivalry^37^. We argue that different paradigms invoking IOC resemble each other on first sight, but upon close inspection also have striking differences: B-CFS reveals how quickly a target in one eye can escape interocular suppression by a mask in the other eye. Hereby, b-CSF taps into mechanisms that propel a hitherto unconscious target into consciousness. As the suppressed stimulus in b-CFS is initially unconscious, an observer cannot consciously favor, attend or otherwise influence processing of the target. BR on the other hand, reveals how two images compete for perceptual dominance for prolonged durations. In this case both images are perceptually dominant in alternation and are therefore subject to top-down processes, like feature-based attention. Note here, that such top-down processes would actually be detrimental for our aim to isolate the effect of VHF on IOC. These kinds of differences might underlie the relatively weak correlation between the outcome measures in BR and b-CFS paradigms in certain experimental contexts. In any case, while there is some debate as to the underlying differences between b-CFS and other IOC-based paradigms, like binocular rivalry (BR)^43,44^, it is clear that b-CFS is at least partly based on IOC. To conclude, we find the effect of VHF on IOC is clear and consistent in b-CFS paradigms: we showed in three ways that the effect of VHF is modulated by IOC *and* we refuted the possibility that a response bias underlay this effect. Yet, the effect of VHF is inconclusive at best in other IOC-based paradigms. One explanation for this discrepancy, could simply be that our study included a much larger amount of data than any other (single) study, thus providing a more robust estimation of the nasotemporal asymmetry. Alternative accounts of this discrepancy might lie in the dependence on specific characteristics of the rivaling stimuli (e.g., relative saliency, eccentricity, etc.), or the outcome measures in specific paradigms (e.g., b-CFS, ongoing rivalry, onset rivalry). Because our present data set does not allow to address these possibilities, we leave it to future research to continue this line of investigation.

Next, we consider three possible functional stages from which the nasal advantage reported here may originate: *before* IOC is resolved, *during* the resolution of IOC, and *after* IOC is resolved. The first stage (*before* OC is resolved) is unlikely to be the source of the nasal advantage as the nasotemporal asymmetries before IOC reflect a qualitatively opposite effect: namely a *temporal* VHF advantage. Examples include studies showing the density of cone-cells is higher in the nasal hemiretina, that visual acuity is better for the temporal VHF, and that saccadic latencies are lower towards temporal VHFs (for review see: Ref.^45^). The last stage (*after* the target has escaped suppression) could theoretically result in a nasal advantage in case observers respond to nasal targets quicker (due to a more liberal response tendency for nasal targets) after the target escaped suppression. However, such a bias for nasal targets is extremely unlikely, as observers are categorically unaware whether a target is presented nasally or temporally. To know whether a target is presented nasally, one must know both its location (left or right) and the eye it is presented to. While judging the location (left or right) is trivial, humans are agnostic as to what eye visual information is presented to^39,40^. It is therefore unlikely that observers have a different response tendency for nasally compared to temporally presented targets. Moreover, we empirically demonstrated that nasally presented targets are reported to be perceived first more often than temporally presented target, when both are presented simultaneously. With the last stage excluded as well, the most probable source of the nasal advantage described here is the stage where IOC is resolved. In line with this, our data showed that differences in IOC (between observers, between eyes of an observer, or between experimental conditions within observers) directly relate to the magnitude of the nasal advantage, solidifying the view that the nasal advantage originates at the stage where IOC is resolved.

**Figure 8:**
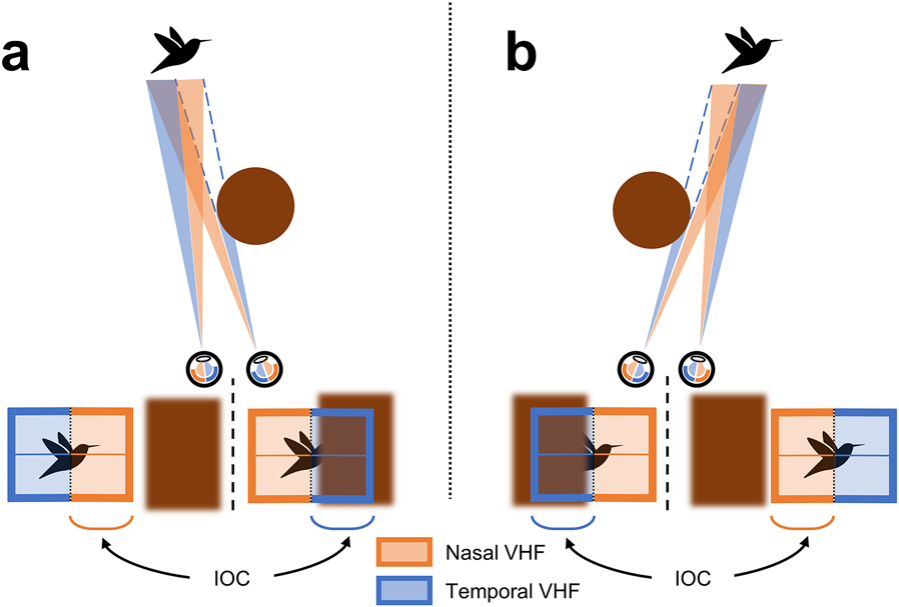
Schematics of the view of a distant object behind an occluder. Panels (**a**) and (**b**) depict two instances where a near object (a brown tree) partly occludes a fixated distant object (a bird), and thereby elicits IOC. These situations illustrate that in both cases, the nasal VHF contains a larger portion of the distant fixated bird than the temporal VHF. The boxes below the left and right eyes (separated by the dashed black lines) represent the same borders (at an arbitrary distance) around the fixation points of the two eyes respectively.

It is tempting to believe that this nasal advantage might serve an adaptive function, given the robustness and size of the effect we have measured. Arnold et al.^46,47^ have argued that resolving interocular competition serves to enhance the visibility of distant fixated objects when (partly) occluded by near objects. Consider the following example: when looking at a bird past a tree, one eye has full view of the bird while the other eye’s view of the bird is (partly) occluded by the tree (see Fig. 8a). Such a situation gives rise to discrepant monocular images, in need of resolution. How does the visual system prioritize the object of interest (the fixated bird) over the object of non-interest (the tree), that compete for interocular dominance? One way in which the visual system can solve this problem is to prioritize stimuli that contain more high-spatial frequencies (the bird, which is fixated and thus yields a sharp retinal image) at the expense of stimuli that contain more low-spatial frequencies (the tree, which is not on the fixated depth plane and thus yields a blurry retinal image). Indeed, high spatial frequency information has more competitive strength than low spatial frequency information in IOC^14,48^. Arnold^47^ notes that, like spatial frequency, many other stimulus characteristics (such as contrast or luminance) determine competitive strength in IOC. Most of these stimulus characteristics that determine competitive strength coincide with stimulus characteristics that differentiate between nearby occluders from distant fixated objects—always in such a way that the distant fixated objects are favored. Likewise, the nasotemporal asymmetry in IOC described in this paper can differentiate between distant fixated objects and nearby occluders. Reconsider the example in which an observer looks at a bird which is partly occluded by a tree (see Fig. 8); assuming that the observer maintains fixation on the (partially) occluded object of interest (i.e., the bird), the nasal VHF will always contain more information about (i.e., a larger portion of) the object of interest, whereas the temporal VHF will contain more information about (i.e., a larger portion of) the nearby occluder. Accordingly, a nasal advantage in IOC could serve to bias perception in favor of a distant object of interest, and to bias perception against the nearby hindering occluder (follow this link: https://8pd765cfbw.cognition.run/ for an interactive demonstration). Notice that whenever an object moves into view from either side, it starts by occluding a temporal portion of the object of interest in one eye. Until the occluder has reached the center of fixation of the eye that is occluded, the other eye’s nasal VHF contains a larger portion of the object of interest. When the occluder moves past the eye’s center of fixation, this eye has no sight on the object in the center of fixation, and other image properties that are known to drive IOC may become more relevant and outweigh the nasal advantage in IOC (e.g., high vs. low spatial frequency, luminance differences, etc.). Taken together, a nasal advantage during IOC can provide a consistent functional benefit in naturalistic vision.

While potentially advantageous in real-world settings, the large VHF asymmetry described here also has important repercussions for researchers using paradigms based on IOC. We advocate to account for the VHF asymmetry in experimental designs based on IOC (see Supplementary Text S4 for further explanations and recommendations as to why, when and how to do it).

In sum, we have shown here that the visual field location in which stimuli are presented, can greatly impact their suppression durations in a b-CFS paradigm. We found that nasally presented stimuli break through interocular suppression faster than temporally presented ones. Moreover, we showed that this asymmetry for visual hemifields is based on interocular competition, as this asymmetry depended on the amount of sensory eye dominance (both within- and between observers), and disappeared in experimental conditions without IOC. We speculate that the advantage for the nasal VHF in IOC could be functional, as distant fixated objects compete from the nasal VHF against nearby occluders in a temporal VHF. Finally, we advocate to account for the RT variance caused by this VHF asymmetry, which is particularly important for designs that are not fully balanced. Above all, we find it fascinating that the seemingly peculiar asymmetry we report on might provide an adaptive advantage in everyday vision, by enhancing the perceptual signal associated with fixated objects of interest.

## Supplementary Information

Supplementary Information.

## Data Availability

We have made all data and all scripts, needed to reproduce our work, publicly available on the Open Science Framework (OSF) platform, accessible thought the link: https://osf.io/e94sw/. The files contain all raw and processed data; as well as the scripts we used to process the data, to compute the reported values, and to create the figures; and finally, the files contain all reported statistical analyses.
