# Supplementary material for: A nasal visual field advantage in interocular competition
Source: Sci Rep. 2022 Mar 17;12:4616. doi: 10.1038/s41598-022-08473-w (PMC8931001; doi:10.1038/s41598-022-08473-w)
Supplement: Supplementary file 1 — Supplementary Information. [file 41598_2022_8473_MOESM1_ESM.pdf]

Supplementary Information

A nasal visual field advantage in interocular competition

Authors: A. Sahakian[1]*, C.L.E. Paffen[1], S. Van der Stigchel[1], S. Gayet[1]

Affiliations:

1. Department of Experimental Psychology & Helmholtz Institute, Utrecht University, Heidelberglaan 1, 3584 CS, Utrecht, the Netherlands

**Corresponding author*: email: a.sahakian@uu.nl

Text S1: Details on exploratory finding

In a previous breaking continuous flash suppression (b-CFS) experiment conducted in our lab[1], we noticed a curious pattern in the data: There was a pronounced response time (RT) difference between (left/right) target locations and, interestingly, this effect was opposite between the eyes (see Supplementary Fig. S1). For example, for targets presented to the left eye (which are masked by CFS in the right eye), the targets right of fixation elicited faster response times than targets left of fixation. The opposite (i.e. faster RT for left than right target locations) seemed the case for targets presented in the right eye. In the experimental trials the targets were always presented to the recessive eye, so that right-eye-dominant observers and left-eye-dominant observers showed an opposite pattern of results.

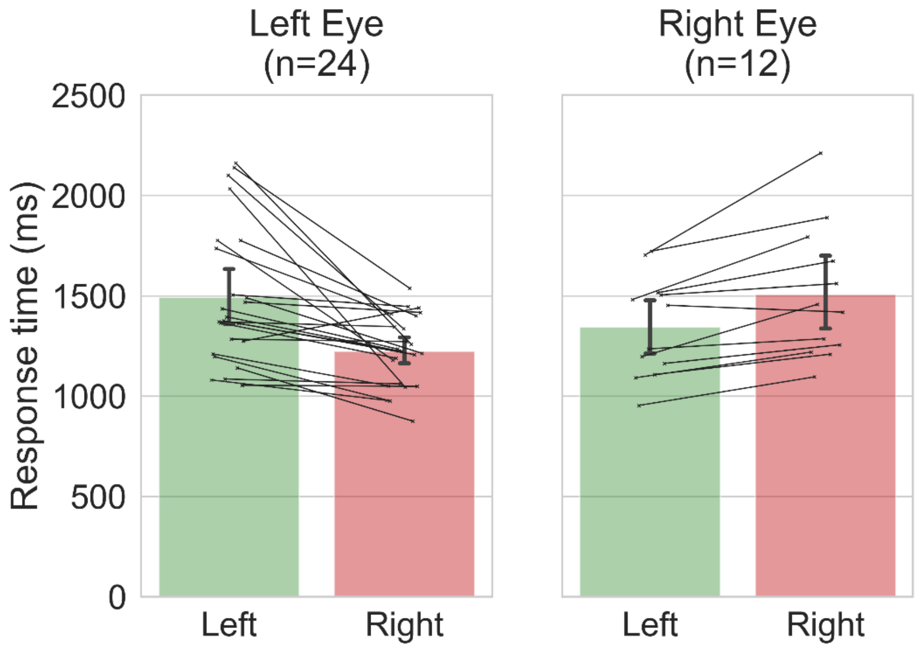

Figure S1: Response times to targets presented to the left and right eyes, left and right from fixation.

The bars represent the group averages, while the lines represent the median response times of individual observers. In this experiment the target was always presented in the recessive eye, and the CFS mask in the dominant eye so to increase response times. Therefore, on the left, only the data of right-eye-dominant observers (n=24) is plotted, while on the right the data of left-eye-dominant observers (n=12) is plotted.

The effect could potentially be generalized in two ways: 1) targets presented in the nasal visual hemifield (VHF) elicit faster response times than targets presented in the temporal VHF; or 2) Targets presented in the same side (e.g. right of fixation) as the eye that is dominant (e.g. the right eye is dominant) elicit faster response times. Although the second hypothesis is somewhat contrived, we could still test which one was true by turning to another part of the data. In order to determine eye dominance each observer completed 15 trials per eye prior to the main experiment. These trials provided us with data for targets presented to both eyes of each observer. If the fist hypothesis was true we would expect to see that nasally presented targets

elicit faster response times in both eyes. If the second was true we would expect that nasally presented targets only elicit faster response times in one eye but not the other.

Visual inspection of the data suggest that in both the dominant and recessive eyes the nasally presented targets elicited faster response times than temporally presented targets (see Supplementary Fig S2). Given that there were only 15 trials per eye (that is about 7 to 8 trials per target location, as location was randomized), it is remarkable that a statistically reliable effect was observed with only 36 observers, testifying to the strength of the RT difference between VHF. Visual inspection of the present data further suggests that the difference between RTs of nasally and temporally presented targets is most pronounced when the target is presented in the recessive eyes of the observers.

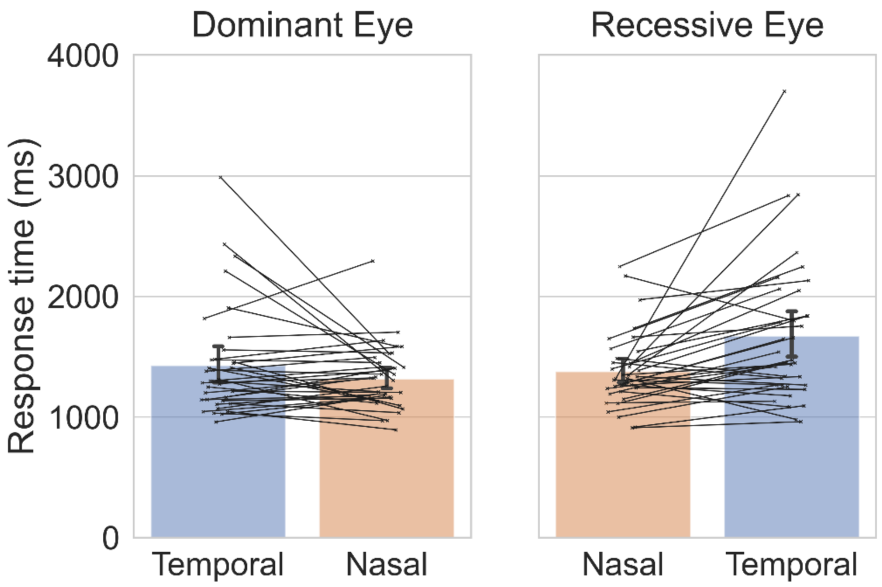

Figure S2: Response times to targets presented to the dominant and recessive eyes, nasally and temporally from fixation.

The bars represent the group averages, while the lines represent the median response times of individual observers. Response times to targets presented to observers' dominant eye are plotted on the left half of the graph, whereas response times to targets presented to observers' recessive eye are presented on the right half of the graph.

References

1. Paffen, C. L. E., Sahakian, A., Struiksma, M. E. & Van der Stigchel, S. Unpredictive linguistic verbal cues accelerate congruent visual targets into awareness in a breaking continuous flash suppression paradigm. *Attention, Perception, Psychophys. 2021 835* 83, 2102–2112 (2021).

Table S1: Extended details on main and supplementary datasets

Dataset	1	2	3	4
**General Descriptives**				
Number of observers	55	181	24	27
Number of trials	128	72	72	288
Trials per eye	64	36	36	144
Balancing	Fully counterbalanced	Fully counterbalanced	Fully counterbalanced	Fully counterbalanced
Effective manipulations	Stimulus size (small vs large), and shape (melon vs box)	N.A.	N.A.	Face vs face part, face emotion (dominant vs non-dominant face)
**Stereoscope Setup**				
Type	Mirror-based	Mirror-based	Mirror-based	Mirror-based
Number of monitors	1	1	2	1
Number of mirrors	2 per eye	2 per eye	1 per eye	2 per eye
Separating LR eye input	Divider (nose to screen)	Small mirror-to-eye distance.	Opposing monitors	Divider (nose to screen)
Effective viewing distance	57 cm	61 cm	55 cm	95 cm
**Monitor Details**				
Model	Benq XL2420Z	Asus PG279Q	Asus PG279Q	Benq XL2420Z
Type	LCD	LCD	LCD	LCD
Refresh rate	120 Hz	144 Hz	120 Hz	120 Hz
**Target Stimuli**				
Appearance (size, shape, color)	2 sizes: 0.85 dva & 1.70 dva, grayscale images of melons and boxes	1.2 dva; circles; red, green, and blue	1.2 dva; rectangular black and with gratings, oriented 45 deg clockwise or counterclockwise.	Grayscale faces (2.2 x 3.6 dva), or rectangular cut-out of eye region (2.2 x 0.8 dva) from these faces
Location(s)	Left or right of fixation	Left or right of fixation (within 30 deg above/below horizontal midline)	Left or right of fixation (within 1.5 dva above/below horizontal midline)	left, right, above or below fixation
Eccentricity	+/- 4.5 dva	1.8 dva	4 dva	+/- 2.6 dva
Presentation time / ramp up time	6 seconds (incl. 1 s ramp up)	Ramp up 1 s	10 seconds (ramp up: 1 s)	16 seconds (ramp up: 1s)
**Masking Stimuli**				
Appearance	Pink noise, rounded to black and white	Pink noise, rounded to black and white	Pink noise, rounded to black and white	Overlapping circles, grayscale
Refresh/flicker rate	10 Hz	10 Hz	10 Hz	10 Hz
Ramp	4 second ramp down (after 1 second)	N.A.	N.A.	N.A.
**Responses**				
Instruction	Report target's location (L/R of fixation) as soon as you can discern it.	Report target's location (L/R of fixation) as soon as you can discern it.	Report target's location (L/R of fixation) as soon as you can discern it.	Report target's location (L/R/U/D of fixation) as soon as you can discern it.
Recording	Keyboard presses: Left and right arrow, or A and D keys. Use index and middle finger of dominant hand.	Keyboard presses: Left and right arrow. Use index and middle finger of right hand.	Keyboard presses: Left and right arrow. Use index and middle finger of right hand.	Keyboard presses: Left, right, up, down arrows.
Response time definition	Target onset until keypress	Target onset until keypress	Target onset until keypress	Target onset until keypress
**Sources**				
Studies with same setup	https://osf.io/thdqb/	https://doi.org/10.1016/j.cognition.2020.104463	https://doi.org/10.1016/j.visres.2020.04.010	10.1037/emo0000550
Studies with same data	N.A.	N.A.	N.A.	10.1037/xge0000521 (Exp 3)

Dataset (continuation)	5	*MC*	*Explr (Exp+Dom)*
**General Descriptives**			
Number of observers	21	19	36
Number of trials	144	128	Exp 1=324, Exp 2=360, Dom=30
Trials per eye	72	64 (monocular), 64 (CFS)	Dom=15,<br>**in Exp all trials in same eye
Balancing	Fully counterbalanced	Fully counterbalanced	Randomized
Effective manipulations	Shape and congruency of shape and visual working memory content	Prevalence of a grating orientation at a specific location (L/R of fixation)	Color congruency of auditory verbal cue.
**Stereoscope Setup**			
Type	Mirror-based	Mirror-based	Mirror-based
Number of monitors	2	1	1
Number of mirrors	1 per eye	2 per eye	2 per eye
Separating LR eye input	Opposing monitors	Inner mirrors	None
Effective viewing distance	55 cm	57 cm	61 cm
**Monitor Details**			
Model	Asus PG279Q	LaCie Electron Blue IV	Asus PG279Q
Type	LCD	CRT	LCD
Refresh rate	120 Hz	100 Hz	144 Hz
**Target Stimuli**			
Appearance (size, shape, color)	Triangles, circles and squares, +/- 1.0 dva	Grayscale Gabor patch (9 cycles per dva; oriented diagonally), circular Gaussian envelope: SD of 1.15 dva.	1.2 dva; circles; red, green, and blue
Location(s)	Left or right of fixation	L/R of fixation, and vertically jittered following a Gaussian distribution (SD = 0.74 dva)	Left or right of fixation (within 45 deg above/below horizontal midline)
Eccentricity	+/- 2 dva	+/- 1.5 dva	1.8 dva
Presentation time / ramp up time	5 seconds (ramp up: 1s)	4 seconds (ramp up: 1s)	8 seconds (ramp up: 1 s)
**Masking Stimuli**			
Appearance	Pink noise, rounded to black and white	Pink noise, rounded to black and white	Pink noise, rounded to black and white
Refresh/flicker rate	10 Hz	10 Hz	10 Hz
Ramp	N.A.	N.A.	N.A.
**Responses**			
Instruction	Report target's location (L/R of fixation) as soon as you can discern it.	Report target's location (L/R of fixation) as soon as you can discern it.	Report target's location (L/R of fixation) as soon as you can discern it.
Recording	Keyboard presses: Left and right arrow. Use index and middle finger of right hand.	Keyboard presses: Left and right arrow. Use index and middle finger of right hand.	Keyboard presses: Left and right arrow. Use right hand.
Response time definition	Target onset until keypress	Target onset until keypress	Target onset until keypress
**Sources**			
Studies with same setup	https://doi.org/10.1016/j.visres.2020.04.010	10.1167/16.11.26, 10.1167/18.3.7	https://osf.io/va46q/
Studies with same data	N.A.	N.A.	https://osf.io/va46q/ (Eye dominance test trials of Exp 1 and Exp 2)

Dataset (continuation)	Two Targets
**General Descriptives**	
Number of observers	19
Number of trials	128
Trials per eye	64
Balancing	Fully counterbalanced
Effective manipulations	Congruency of color and visual working memory content
**Stereoscope Setup**	
Type	Mirror-based
Number of monitors	1
Number of mirrors	2 per eye
Separating LR eye input	Divider (nose to screen)
Effective viewing distance	57 cm
**Monitor Details**	
Model	LaCie Electron Blue IV
Type	CRT
Refresh rate	100 Hz
**Target Stimuli**	
Appearance (size, shape, color)	1.08 dva; circles; red, green, blue and purple
Location(s)	Random angular position on the left and right arcs of an imaginary circle, delimited by its main diagonals
Eccentricity	1.62 dva
Presentation time / ramp up time	10 seconds (ramp up: 1 s)
**Masking Stimuli**	
Appearance	Pink noise, rounded to black and white
Refresh/flicker rate	10 Hz
Ramp	N.A.
**Responses**	
Instruction	Report target's location (L/R of fixation) as soon as you can discern it.
Recording	Keyboard presses: Left and right arrow/.
Response time definition	Target onset until keypress
**Sources**	
Studies with same setup	https://doi.org/10.1167/16.11.26
Studies with same data	https://doi.org/10.1167/16.11.26

Text S2: Details on Monocular Control Dataset

To confirm in one more experimental way whether the nasal advantage we have reported on related to interocular competition (IOC) specifically, we included an additional analysis. We could turn to a dataset which was retrieved from a student project supervised by author SG[1]. In this breaking continuous flash suppression (b-CFS) experiment observers responded to targets which were presented to both eyes and to both VHFs, and, crucially for our cause, there were two target presentation conditions: 1) a 'binocular' (conventional b-CFS) condition where the target was presented to one eye and the CFS mask to the other eye, and 2) a 'monocular' condition where both the target and the mask were presented to one eye (the targets contrast was slowly ramped up superimposed on the CFS mask; see Supplementary Fig. S5). These two conditions allowed us to check for the presence of a nasal advantage in a situation where targets were interocularly suppressed, and a situation where targets were not interocularly suppressed.

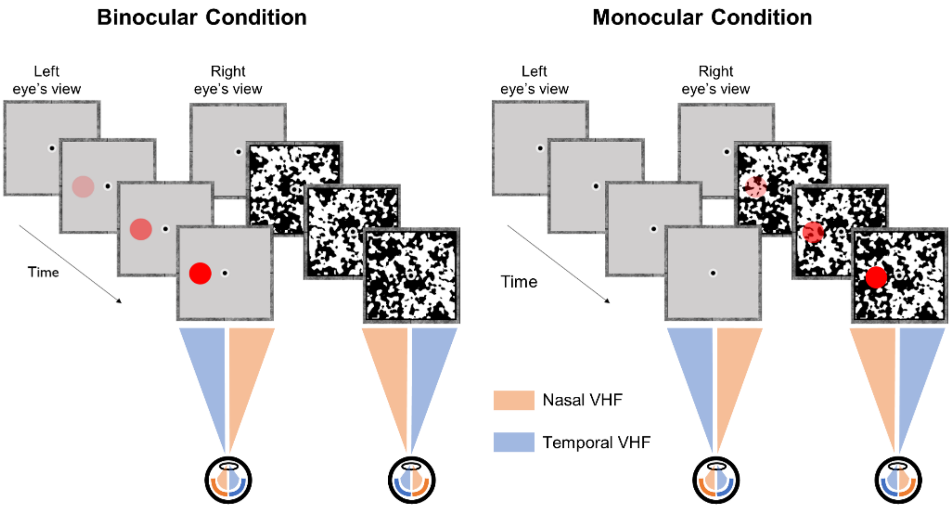

Figure S5: Schematic depiction of a binocular and monocular b-CFS paradigm.

On the left, one trials of a binocular condition is shown, this is how a conventional b-CFS trial looks like: a target is gradually introduced to one eye, while the other eye is presented with a dynamic mask until the target is reported. On the right a monocular condition is depicted, where the only difference is that the target is gradually introduced to the *same eye* as the eye which is presented with the dynamic mask. As opposed to the binocular condition, in the monocular condition the target is not interocularly suppressed by the mask.

This dataset was standardized as described in the main manuscript, taking into account the presentation type (binocular or monocular) of the target stimulus. Specifically, this entailed that RTs were normalized by the presentation type as well as observer and eye of target presentation as in the other datasets.

As we were interested in the effect size of the nasal advantage in the two conditions we performed a Bayesian RM ANOVA with factors ‘presentation type’ (binocular & monocular) and ‘VHF’ (nasal & temporal). The BF in favor of the presence of an interaction was 4.33. Subsequent directional Bayesian paired samples *t*-tests showed that in the binocular condition it was 3.64 times more likely that the nasal targets elicited faster RTs than temporal targets, but in the monocular condition, it was only 0.731 times more likely that the nasal targets elicited faster RTs. Note that the stronger nasal advantage in the IOC condition (compared to the monocular condition) cannot be explained by a floor effect in the monocular condition diminishing any detection time difference, as the study's original effect-of-interest (described in *ref.*[1]) was at least as (if not more) reliable in the monocular condition than in the IOC condition.

These results show that the advantage nasally presented targets have over temporally presented targets in response time, is present only when targets are interocularly suppressed. This finding suggests that origin of the nasal advantage we report on lies in the mechanisms resolving IOC.

References:

1. Blom, T. & Gayet, S. Visual Statistical Learning Leads to More Efficient Decision Processes, not to Prioritization for Access to Visual Awareness. (2015).

Text S3: Two simultaneous targets Dataset

We interpreted the faster response times for targets presented in the nasal visual hemifield (VHF) compared to the temporal VHF as evidence that the nasal VHF has more competitive strength in interocular competition than the temporal VHF. Here, we consider the possibility that nasal and temporal targets were *perceived* equally fast (and thus had equal competitive strength), but that nasally presented targets somehow evoked faster response times.

To test whether nasally presented targets were actually perceived before temporally presented targets, we turned to one more breaking continuous flash suppression (b-CFS) experiment with an important adaptation in its design[1]. In this experiment, *two* targets are introduced simultaneously, one in each VHF (see Supplementary Fig. S4). Participants were instructed to report *where* (left or right) they saw a target appear first. With this adapted paradigm the outcome measure includes a response choice, reflecting which target was perceived first, regardless of response speed. Consequently, if the nasal advantage reflects a difference in detection times (rather than perception-unrelated response speed), we expect observers to report nasally presented targets to appear first more frequently than temporally presented targets. If the nasal advantage reported in the main manuscript was caused by a difference in response speed arising after interocular competition (IOC) is resolved, however, we would expect nasally and temporally presented targets to be reported to appear first equally often.

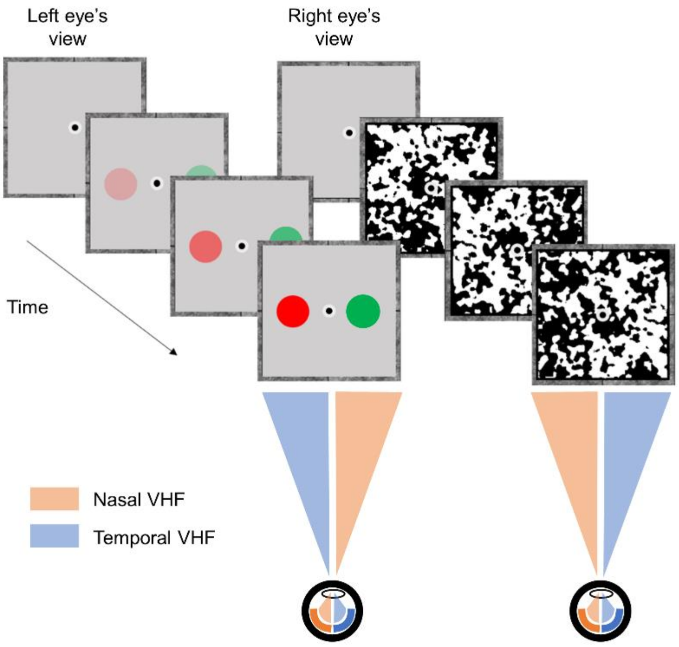

Figure S4: Schematic overview of b-CFS task with two targets

Two targets (one left and one right of fixation) are gradually introduced to one eye, while high-contrast patterns are flashed (at ~10 Hz) to the other eye. As the patterns are much more salient than the targets, the targets will initially be suppressed from consciousness. Typically one of the targets will break though suppression (and become visible) sooner than the other. Observers have to report the location (left or right) of the target that appears first, as fast as possible. The reported location provides, besides a response time measure, a temporal order judgement measure: namely, which target broke through suppression first.

Nineteen observers successfully completed the experiment which consisted of 128 trials. For three observers a number (16, 5 and 9 respectively) of trials was excluded, as the response times were either too fast or too slow (i.e., outside the time window of 0.35 to 10 seconds). The eye to which the target was presented was randomized, but balanced within observers.

For each observer we counted how many of the first reported targets were presented in the nasal VHF. We then computed what proportion this was of the total number of trials of the observer. We found that on average observers reported to first perceive the nasal target in 66% (SD = 20%) of trials (see Supplementary Fig. S5). Naturally, in the other 34% trials the temporal target was reported to appear first. To test the hypothesis that the proportion of nasal-first reports were higher than chance (0.5) we performed a Bayesian directional one-

sample *t*-test across participants. We found strong evidence (BF_{10} = 34.3) in favor of our hypothesis that nasal targets were more often perceived first.

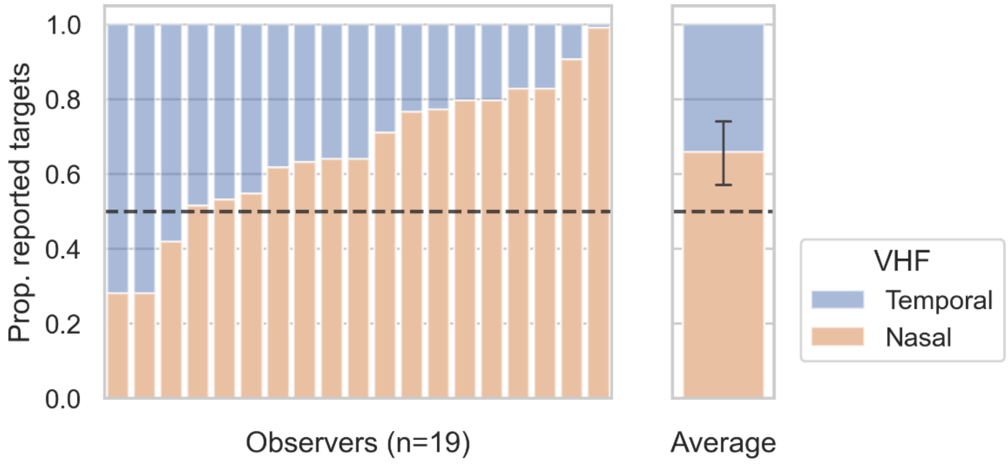

Figure S5: Proportion reported first perceived target locations.

The proportion of reported targets which happened to be in a nasal or temporal visual hemifield (VHF) for each observer (sorted). The bar on the left shows the average proportion across all observers. The dotted line represents the chance level, which was 0.5 as the two targets were always presented simultaneously. The error bar represents the bootstrapped 95% confidence interval.

These finding show that stimuli in presented in the nasal VHF are *perceived* earlier than targets presented in the temporal VHF, and thus indeed break though suppression faster. We want to stress that observers were presumably completely unaware of the fact that they are choosing nasal targets more often than temporal targets. From the observers' perspective, they were only indicating whether a target first appeared left or right of fixation. Because observers could not know – nor directly perceive – *to which eye* the targets were presented (and to which the mask), they could not distinguish temporally from nasally presented targets (e.g., left in left eye versus left in right eye). To conclude, we empirically demonstrated that the nasal advantage in b-CFS is based on a perceptual difference, and not merely based on a response bias.

References:

1. Gayet, S., Maanen, L. van, Heilbron, M., Paffen, C. L. E. & Stigchel, S. Van der. Visual

input that matches the content of visual working memory requires less (not faster) evidence sampling to reach conscious access. *J. Vis.* 16, 26–26 (2016).

Text S4: Recommendations for unbalanced datasets

We demonstrated that the location at which a target is presented in a b-CFS experiment affects the target's suppression time greatly. This difference in suppression times was several times larger even, than the effect of interest as reported in many published b-CFS studies[1–3]. Stein and colleagues, for example, investigated the effect of the dominance of a face on the breakthrough times in a b-CFS paradigm[2]. They found a significant difference between suppression times for dominant and not-dominant faces. This effect remained significantly present even when the target stimuli were upside-down cut-out regions of the eyes (of the original face stimuli) or upside down contrast reversed cut-out regions of the eyes (see Supplementary Fig. S6).

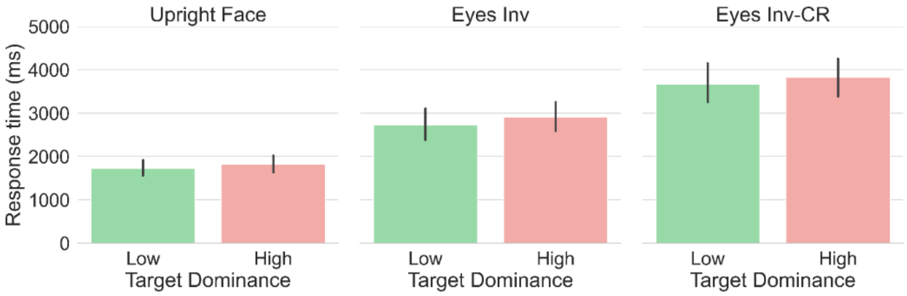

Figure S6: Response times to stimuli classes with high and low dominance ratings. The response times in a b-CFS study where the target was either an upright face, an upside-down cutout portion of the eyes, or a contrast reversed version of the upside-down cutout portion of the eyes. Each face (or cut-out of a face) could either have a high or low facial dominance rating. The error bars represent bootstrapped 95% confidence intervals.

However when we split the data into trials where the target was in the nasal visual hemifield (VHF) and trials where the target was in the temporal VHF, it is apparent that the difference in RTs between target locations is larger than the difference in high and low dominance of the faces (see Supplementary Fig. S7). This example shows important implications for researchers implementing the b-CFS paradigms. If for some reason the targets stimuli are not balanced over the nasal and temporal VHF locations – either by design or due to trial exclusion– the effect of VHF location can potentially skew the results. This could lead to not finding a sought after effect, or, arguably worse, falsely identifying a non-existent effect.

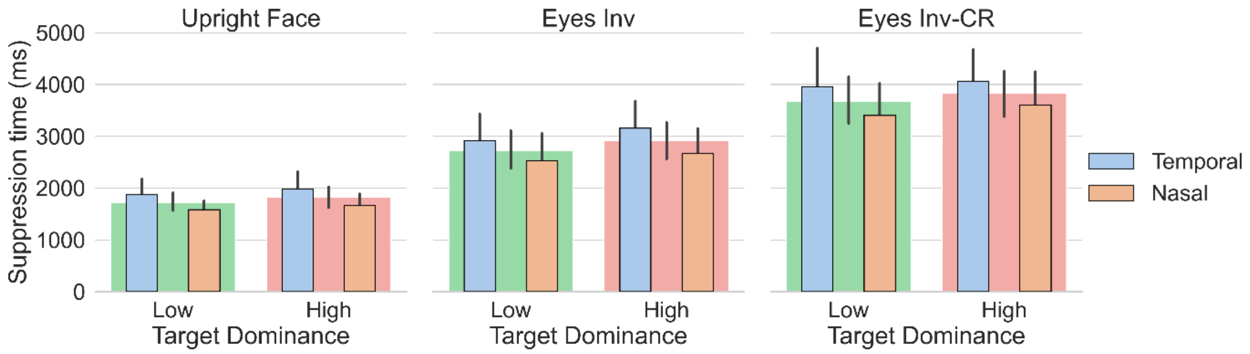

Figure S7: Response times per VHF to stimuli classes with high and low dominance rating. The same response time data as shown in Supplementary Fig. S6, but now the response times for targets *within each condition* are split over nasal and temporal VHF of target presentation. The effect of VHF of target presentation on RTs within a condition is more pronounced than that of facial dominance, which was one of the sought-after effects. Removing the large variance due to the VHF (a factor of non-interest) could be beneficial for statistical analyses of other factors of interest.

Our recommendation to account for this unwanted variance in the data is to normalize the response times over VHF. The benefits of normalizing data have been demonstrated before by Gayet and Stein[4]. In line with their method we propose the following extension of normalizing response time data. This is specifically useful for b-CFs experiments with lateralized targets.

The procedure consist of dividing each RT to targets in a given eye and a given VHF, by the mean of all RTs in that eye and VHF. Or in a stepwise approach:

1. Group each observer's trials by eye and VHF of target presentation.
2. Determine the mean of each group of trials.
3. Divide each RT in a trial by the mean of the group it belongs in.

This normalization procedure will remove unwanted variance (i.e. variance *not* caused by the sought-after effect) due to overall RT differences between observers, RT differences between dominant and recessive eyes (between and within observers), and RT differences between nasal and temporal VHF location (between and within observers).

References

1. Gayet, S., Paffen, C. L. E. & Van der Stigchel, S. Information Matching the Content of Visual Working Memory Is Prioritized for Conscious Access. *Psychol. Sci.* 24, 2472–2480 (2013).

2. Stein, T., Awad, D., Gayet, S. & Peelen, M. V. Unconscious processing of Facial Dominance: The role of low-level factors in access to awareness. *J. Exp. Psychol. Gen.* 147, e1–e13 (2018).

3. Gayet, S., Van Der Stigchel, S. & Paffen, C. L. E. Breaking continuous flash suppression: Competing for consciousness on the pre-semantic battlefield. *Frontiers in Psychology* vol. 5 460 (2014).

4. Gayet, S. & Stein, T. Between-subject variability in the breaking continuous flash suppression paradigm: Potential causes, consequences, and solutions. *Front. Psychol.* 8, 437 (2017).
